# Nation-wide survey of screening practices to detect carriers of multi-drug resistant organisms upon admission to Swiss healthcare institutions

**DOI:** 10.1186/s13756-019-0479-5

**Published:** 2019-02-13

**Authors:** Romain Martischang, Niccolo Buetti, Carlo Balmelli, Mirko Saam, Andreas Widmer, Stephan Harbarth

**Affiliations:** 1 Infection Control Programme and WHO Collaborating Centre on Patient Safety, University of Geneva Hospitals and Faculty of Medicine, Geneva, Switzerland; 2 Department of Infectious Diseases, Bern University Hospital, Bern, Switzerland; 3 Servizio di Prevenzione Delle Infezioni e Medicina del Personale, Ente Ospedaliero Cantonale, Ticino, Switzerland; 4 Communication in Science, Geneva, Switzerland; 5 Department of Infectious Diseases and Infection Control, University Hospital Basel, Basel, Switzerland

**Keywords:** Infection prevention and control, Contact isolation, Admission screening, Enterobacteriaceae, Multi-drug resistant, Carbapenem-resistant, Extended-spectrum beta-lactamase, Cross-infection, Survey

## Abstract

As emergence and spread of multi-drug resistant organisms (MDRO) requires a standardized preventive approach, we aimed to evaluate current MDRO admission screening practices in Swiss hospitals and to identify potential barriers impeding their implementation. In early 2018, all Swiss public and private healthcare institutions providing inpatient care were contacted with a 34-item questionnaire to investigate current MDRO admission screening policies. Among 139 respondents representing 180 institutions (response rate, 79%), 83% (149) of institutions implemented MDRO admission screening, while 28% of private and 9% of public institutions did not perform any screening. Targeted high-risk screening included carbapenemase producers, extended-spectrum beta-lactamase producers and methicillin-resistant *Staphylococcus aureus* at the institutional level for respectively 78% (115), 81% (118) and 98% (145) of screening institutions. Vancomycin-resistant enterococci (44% of institutions), multi-resistant *Acinetobacter baumanii* (41%) and *Pseudomonas aeruginosa* (37%) were systematically searched only by a minority of screening institutions. A large diversity of risk factors for targeted screening and some heterogeneity in body sites screened were also observed. Admission-screening practices were mostly impeded by a difficulty to identify high-risk patients (44%) and non-compliance of healthcare workers (35%). Heterogeneous practices and gaps in small and privately-owned institutions, as well as a mismatch between current epidemiologic MDRO trends and screening practices were noticed. These results highlight the need for uniform national MDRO screening standards.

**Electronic supplementary material:**

The online version of this article (10.1186/s13756-019-0479-5) contains supplementary material, which is available to authorized users.

## Introduction

Early detection of multi-drug resistant organisms (MDRO) carriage upon admission could allow timely implementation of infection control measures and the appropriate selection of empiric antimicrobial therapy [1]. Few nationwide surveys investigated real-life MDRO screening practices upon admission [2–5]. In 2010, an unpublished survey conducted in Swiss intensive care units (ICUs) revealed heterogeneous MDRO screening practices. Endemicity among MDROs in Switzerland differs according to community or hospital settings. ESBL-producing *Escherichia coli* is considered as endemic in the general population, especially in the institutionalized elderly (extended-spectrum beta-lactamase (ESBL) *E.coli* prevalence of 22% among clinical isolates from nursing homes in 2017) [6], whereas acute care hospitals also consider methicillin-resistant *Staphylococcus aureus* (MRSA) - despite decreasing trends - (prevalence of 8% among clinical *S. aureus* isolates in 2014) [7] and ESBL-producing *Klebsiella pneumoniae* as endemic (7.7% of ESC-R invasive isolates in 2017) [8]. The emergence and spread of MDRO requires a standardized preventive approach on a national scale. We therefore evaluated current MDRO admission screening practices in Swiss hospitals and identified potential barriers impeding their implementation.

## Methods

From January to March 2018, a nation-wide 34-item questionnaire was sent to 228 Swiss public and private healthcare institutions providing inpatient acute care. Psychiatric institutions, nursing homes, palliative care and pain therapy centers were excluded. Three reminders as well as a phone call were addressed to each non-responding institution.

The survey was translated in the three official languages, pre-tested locally and shared through the online platform SurveyMonkey® (see French and German versions of the Online Survey, Additional files 1 and 2). We collected information about the characteristics of each hospital, in addition to current practices concerning universal and targeted MDRO screening for patients at-risk at admission, risk factors considered for targeted screening, body sites for sampling swabs and cultures, preemptive contact precautions for high-risk patients, the presence of local guidelines and problems faced to implement on-admission screening.

All analyses were institution-based (*n* = 180) and not respondent-based (*n* = 139), since some respondents were in charge of several institutions. Data were extracted from the online platform to an Excel® spread-sheet, checked for accuracy and exported for descriptive analysis using STATA 15.0® (StataCorp LLC, College Station, TX).

## Results

Overall, 139 respondents, mainly nurses (56%) and physicians (37%) replied for 180 institutions (response rate, 79%), with 57% from public institutions and 61% from small-size (< 200 beds), 21% medium-size, and 18% large-size institutions (> 500 beds). All non-responders were small-size institutions. The majority of hospitals (72%) was located in the Swiss-German part. Eighty-three percent of institutions (149) implemented some type of MDRO admission screening, while 28% of private and 9% of public institutions did not perform any screening. Universal MRSA screening of all admitted patients was not performed on an institutional level by any hospital, except for a few specific units in 6% of screening institutions. Targeted high-risk screening at the institutional level included carbapenemase-producing *Enterobacteriaceae* (CPE), ESBL-producing *Enterobacteriaceae* and MRSA, which were monitored by 78% (*n* = 115), 81% (*n* = 118) and 98% (*n* = 145) of hospitals, respectively (Table 1). Vancomycin-resistant enterococci (VRE) (44%), multi-resistant *Acinetobacter baumanii* (41%) and *Pseudomonas aeruginosa* (37%) were systematically searched only by a minority of institutions with on-admission screening programs, without differences between small and large institutions.

**Table 1:** Targeted high-risk MDRO screening among public and private hospitals in Switzerland

	ESBL	CPE	MDR-*Acinetobacter*	MDR-*Pseudomonas*	VRE	MRSA
Targeted screening (%)						
Public (*n* = 102)^a^:						
Institutional:	82 (89%)	77 (83%)	37 (40%)	36 (39%)	38 (41%)	93 (100%)
Only in certain units:	0	0	1 (1%)	2 (2%)	4 (4%)	0
None:	10 (11%)	16 (17%)	55 (59%)	55 (59%)	51 (55%)	0
Private (*n* = 78)^b^:						
Institutional:	36 (67%)	38 (70%)	23 (43%)	18 (33%)	27 (50%)	52 (95%)
Only in certain units:	4 (7%)	4 (8%)	2 (4%)	8 (15%)	3 (6%)	3 (5%)
None:	14 (26%)	12 (22%)	28 (53%)	28 (52%)	24 (44%)	0

*Abbreviations*: *ESBL* extended-spectrum beta-lactamase, *CPE* carbapenemase-producing enterobacteriaceae, *MDR* multi-drug resistant, *VRE* vancomycin-resistant *enterococcus*, *MRSA* methicillin resistant *Staphylococcus aureus*

^a^Missing values for: *ESBL = 10, CPE = 9, Acinetobacter baumanii = 9, Pseudomonas aeruginosa = 9, VRE = 9 and MRSA = 9*

^b^Missing values for: *ESBL = 24, CPE = 24, Acinetobacter baumanii = 25, Pseudomonas aeruginosa = 24, VRE = 24 and MRSA = 23*

Frequently used risk factors to screen patients considered at high risk for MDRO carriage were “known carriers”, “hospitalization abroad” and a “direct transfer from abroad” (Table 2). Other risk factors are heterogeneously recognized among institutions. Of note, few hospitals (19%) systematically screen patients who have been transferred from other Swiss hospitals for VRE carriage, despite increasing VRE rates and ongoing outbreaks in Switzerland.

**Table 2:** Patient-level risk factors considered for targeted MDRO screening upon admission

	ESBL (*n* = 122)	CPE (*n* = 119)	MDR-Acinetobacter (*n* = 62)^a^	MDR-Pseudomonas (*n* = 63)^a^	VRE (*n* = 72)	MRSA (*n* = 148)
	(*n* = number of centers performing a targeted screening for each pathogen)					
Risk factors used for targeted admission screening (%)						
Known MDRO patient:	111 (91%)	111 (93%)	59 (95%)	60 (95%)	67 (93%)	143 (97%)
Direct transfer from abroad:	114 (93%)	107 (90%)	41 (66%)	37 (59%)	54 (75%)	144 (97%)
Direct transfer from Switzerland^b^:	33 (27%)	29 (24%)	13 (21%)	14 (22%)	14 (19%)	71 (48%)
Transfer from a long term care facility:	11 (9%)	7 (6%)	3 (5%)	4 (6%)	5 (7%)	32 (22%)
Hospitalization abroad in the recent past^c^:	103 (84%)	98 (82%)	37 (59%)	32 (51%)	47 (65%)	109 (74%)
Travel in a country with endemic MDRO:	28 (23%)	34 (29%)	16 (25%)	18 (29%)	19 (26%)	35 (24%)
Other:	38 (31%)	41 (34%)	23 (37%)	21 (33%)	21 (29%)	84 (57%)

*Abbreviations*: *ESBL* extended-spectrum beta-lactamase, *CPE* carbapenemase-producing enterobacteriaceae, *MDR* multi-drug resistant, *VRE* vancomycin resistant *enterococcus*, *MRSA* methicillin resistant *Staphylococcus aureus*

^a^Missing values for: MDR *Acinetobacter baumanii = 1, MDR Pseudomonas aeruginosa = 1*

^b^Mainly Western Switzerland and Tessin were targeted when considering a direct transfer from Switzerland

^c^Varying timeframes considered as recent past, mainly from 6 to 12 months

Heterogeneity subsists on the choice of body site sampling. Nares (99%), throat (81%) and inguinal sampling (91%) are leading body sites to screen for MRSA, whereas anal or rectal swabs are most frequently used for ESBL (89%), CPE (94%) or VRE (88%) screening. However, in some centers, inguinal screening was also performed for enteric bacteria. For MDR-*A. baumanii* and *P. aeruginosa*, a large variety of body sites were screened (anal, rectal, inguinal, throat or nasal swabs)*.* For high-risk patients, only 23% (33/142) of hospitals routinely performed repeat swabs in case of one negative screening result. A total of 90% (86/96) of ICUs implemented pre-emptive contact precautions, including placement in a single room in 63% of ICUs.

Despite local recommendations for admission screening provided by 96% (137/142) of hospitals, these practices were mostly impeded by a difficulty to identify high-risk patients (44%) and non-compliance of healthcare workers (35%). Reimbursement issues were less commonly cited as an obstacle (15%).

## Discussion

This nation-wide survey to examine current practices of MDRO admission screening was answered by 180 institutions, representing an excellent response rate and the diversity of healthcare institutions in Switzerland, among public and private institutions of different sizes. This survey revealed good compliance with on-admission MDRO screening practices in larger acute-care hospitals, but also important gaps in small and private institutions.

This survey differs from previous national surveys evaluating MDRO screening practices at admission, mainly because of its higher response rate and the reporting of both risk factors and body sites sampled according to MDRO species [2–5]. Only one national survey performed in France in 2012 addressed public and private healthcare facilities. This survey observed that only 34% of 286 institutions reported management of patients at-risk at the time of admission [3].

A mismatch between the current epidemiologic situation and screening practices was noticed with a disproportionate focus on MRSA (in particular in patients transferred from the French and Italian speaking parts of Switzerland) and a lack of awareness of possible spread of *A. baumanii*, *P. aeruginosa* and VRE by unknown carriers, including patients transferred within Switzerland. Indeed, nosocomial MRSA incidence has been declining, whereas VRE rates are rapidly increasing [7, 9, 10]. In addition, severe nosocomial outbreaks of *A. baumanii* infections linked to imported cases have occurred in Switzerland in the past [11]. Therefore, targeted high-risk screening should also include other MDROs beside MRSA.

A recent travel history to foreign countries without hospitalisation was rarely used as a risk factor to define high-risk patients eligible for screening at admission (23–29% of institutions according to the type of MDRO). This policy concerned in particular South-Asian countries with hyperendemic MDRO occurrence, such as India, Pakistan, Bangladesh, Nepal and Sri Lanka. A recent travel history to North America or U.S. citizenship were not considered as risk factors by any Swiss institution, despite increasing importation of community MRSA into Switzerland [12].

Heterogeneity was also observed among risk factors considered for targeted screening, probably due to a lack of national consensus on multiple criteria supporting surveillance programs. Adding to this complexity, actual controversies addressing admission screening policies support the requirement for updated and uniform standards: species to be screened, risk factors considered for targeted screening, number of screening swabs to be performed at admission, among others. Interestingly, cost considerations did not play an important role in implementing MDRO screening policies.

This survey has limitations. First, we were unable to perform external validation of the respondents’ answers. Second, this survey did cover neither screening practices beyond the admission procedure nor variability in MDRO control measures or laboratory detection methods. Third, the design of the study did not allow correlating MDRO screening practices to nosocomial MDRO transmission rates.

In summary, these results highlight the need for uniform national MDRO screening standards. It also demonstrates a lack of awareness about current MDRO trends, focusing on MRSA rather than VRE or gram-negative MDROs, and ongoing confusion about risk factors that might be addressed through uniform national standards. Harmonized, clear and accessible guidelines – which are already available in some countries – could support standardization of risk factors used for targeted admission screening and of sample sites for admission screening [13, 14].

## Additional files

Additional file 1: Online survey French. (PDF 349 kb)

Additional file 2: Online survey German. (PDF 348 kb)

## Abbreviations

CPE: Carbapenemase Producing *Enterobacteriaceae*
ESBL: Extended-Spectrum Beta-Lactamase
ICU: Intensive care unit
MDRO: Multi-Drug Resistant Organism
MRSA: Methicillin-Resistant *Staphylococcus aureus*
VRE: Vancomycin-Resistant *Enterococcus*
