# Supplementary material for: Nation-wide survey of screening practices to detect carriers of multi-drug resistant organisms upon admission to Swiss healthcare institutions
Source: Antimicrob Resist Infect Control. 2019 Feb 13;8:37. doi: 10.1186/s13756-019-0479-5 (PMC6375162; doi:10.1186/s13756-019-0479-5)
Supplement: Supplementary file 1 — Online survey French. (PDF 349 kb) [file 13756_2019_479_MOESM1_ESM.pdf]

Pratiques de dépistage des bactéries multirésistantes à l'admission.

Enquête auprès des hôpitaux suisses.

Schweizerische Eidgenossenschaft
Confédération suisse
Confederazione Svizzera
Confederaziun svizra

Département fédéral de l'intérieur DFI
Office fédéral de la santé publique OFSP

Stratégie Antibiorésistance StAR

SGM·SSM

swissnoso
National Center for Infection Control

HUG Hôpitaux Universitaires Genève

Instructions:

- Ce questionnaire doit être rempli par une personne sensibilisée au contrôle des infections dans votre établissement.

- La durée de ce questionnaire ne dépassera pas 15 min.

- Votre rôle est déterminant pour finaliser les recommandations suisses pour le dépistage des bactéries multirésistantes à l'admission.

Nous vous prions de répondre à toutes les questions.
Vos réponses sur ce questionnaire seront totalement confidentielles.
Si vous avez des questions ou besoin d'une assistance<br>pour remplir ce questionnaire, prière de nous contacter:
Personne de contact: Romain Martischang
Téléphone: 022 372 98 97<br>Lundi - Vendredi<br>08:30 – 17:30
Adresse e-mail: romain.martischang@hcuge.ch
Votre participation est essentielle pour le succès de ce projet !
MERCI BEAUCOUP POUR VOTRE SOUTIEN

Pratiques de dépistage des bactéries multirésistantes à l'admission.

Enquête auprès des hôpitaux suisses.

Informations additionnelles:

Consignes pratiques:

Pour avancer dans ce questionnaire, cliquez sur "Suiv.", pour retourner en arrière, cliquez sur "Préc." Veuillez ne pas utiliser les boutons fléchés de votre navigateur.

Si possible, remplir ce questionnaire en une fois. Toutefois, vos réponses seront sauvegardées à chaque fois qu'une nouvelle page s'affichera. Vous avez ainsi la possibilité de fermer ce questionnaire pour le reprendre plus tard, sous réserve des conditions suivantes:
1) Utiliser le même ordinateur et le même navigateur (google chrome, internet explorer...) pour reprendre ce questionnaire.
2) Ne pas vider l'historique de votre navigateur.
(Le cache avec l'ensemble des cookies enregistrés sur votre navigateur.)

Abréviations utilisées:

- BMR *: bactérie multirésistante.*
- E-BLSE *: Entérobactéries productrices de Beta-Lactamase à Spectre Elargi (Inclus particulièrement Klebsiella et/ou E.coli producteurs de BLSE).*
- CPE*: Entérobactéries productrices de Carbapénémases.*
- MDR-Acinetobacter ou MDR-Pseudomonas*: Acinetobacter ou Pseudomonas spp résistants au minimum à un agent parmi 3 classes antibiotiques ou plus.*
- MRSA*: methicillin-resistant Staphylococcus aureus.*
- VRE*: vancomycin-resistant Enterococcus.*

Pratiques de dépistage des bactéries multirésistantes à l’admission.

Enquête auprès des hôpitaux suisses.

Données épidémiologiques

* Données personnelles:

Nom, Prénom:

Adresse e-mail:

* Votre fonction:

* Données institutionnelles:

Nom de l'hôpital:

Ville:

Canton:

* Catégorie de l'hôpital:

○ Public ○ Privé

* Secteurs de soins hospitaliers proposés par votre institution:

(Plusieurs réponses possibles.)

☐ Soins aigus

☐ Soins chroniques et réhabilitation

☐ Pédiatrie

☐ Psychiatrie

☐ Autre:

* Combien de lits destinés aux soins aigus avez-vous dans votre institution ?

○ < 200 ○ 200 - 500 ○ > 500 ○ Je ne sais pas

* Présence d'une unité de soins intensifs (USI):

(Les unités de soins intermédiaires ne sont pas comptées comme des unités de soins intensifs)

○ Oui ○ Non

* Pratiquez-vous le dépistage à la recherche d'au moins une classe de BMR à l'admission dans votre hôpital ?

(Que ce soit de façon universelle, sur chaque patient, ou bien ciblée selon un profil à risque.)

◯ Oui ◯ Non

Pratiques de dépistage des bactéries multirésistantes à l’admission.

Enquête auprès des hôpitaux suisses.

Dépistage universel à l'admission

* Y a-t-il certaines unités dans votre hôpital pratiquant le dépistage universel à l'admission pour les BMRs comme test de routine, sur chaque patient hospitalisé et indépendamment d'un profil à risque particulier?

	Oui	Non	Je ne sais pas
USI seulement	◯	◯	◯
Autre(s) unité(s) *(Chirurgie, gériatrie...)*	◯	◯	◯

Si d'autres unités sont concernées, veuillez les nommer:

Pratiques de dépistage des bactéries multirésistantes à l’admission.

Enquête auprès des hôpitaux suisses.

Dépistage universel à l'admission

* Quelle(s) BMR(s) dépistez vous de manière universelle à l'admission ?

	E-BLSE	CPEs	MDR-Acinetobacter	MDR-Pseudomonas	VRE	MRSA
USI seulement	☐	☐	☐	☐	☐	☐
Autre(s) unité(s) *(Chirurgie, gériatrie...)*	☐	☐	☐	☐	☐	☐

Pratiques de dépistage des bactéries multirésistantes à l’admission.

Enquête auprès des hôpitaux suisses.

Dépistage ciblé à l'admission

* Pratiquez-vous un dépistage ciblé à l’admission pour une ou plusieurs BMR(s), en fonction du profil de risque des patients hospitalisés ?

Les écouvillons de dépistage sont ciblés lorsqu’ils sont utilisés sur les patients avec des facteurs de risque uniquement.

◯ Oui ◯ Non ◯ Je ne sais pas

Pratiques de dépistage des bactéries multirésistantes à l’admission.

Enquête auprès des hôpitaux suisses.

Dépistage ciblé à l'admission

1. E-BLSE

* Pratiquez-vous un dépistage ciblé à l’admission pour les E-BLSE, en fonction du profil de risque des patients hospitalisés ?

Les écouvillons de dépistage sont ciblés lorsqu’ils sont utilisés sur les patients avec des facteurs de risque uniquement.

◯ Oui, dans toute l'institution ◯ Non ◯ Je ne sais pas

◯ Oui, dans une ou plusieurs unité(s):
(ex: USI, unités de dialyse...)

Pratiques de dépistage des bactéries multirésistantes à l’admission.

Enquête auprès des hôpitaux suisses.

Dépistage ciblé à l'admission

2. E-BLSE

* Quels sont les facteurs de risque considérés pour le dépistage à l'admission des E-BLSE?

- [ ] Je ne sais pas
- [ ] Porteur déjà connu pour une E-BLSE
- [ ] Transfert direct venant d'un hôpital à l'étranger
- [ ] Transfert direct venant d'un hôpital Suisse
- [ ] Transfert direct venant d'un établissement de soin à longue durée ou d'une maison de retraite en Suisse
- [ ] Antécédent d'hospitalisation récente à l'étranger (sans transfert direct)
- [ ] Antécédent de voyage dans un pays endémique pour les BMRs
- [ ] Si autre(s) facteur(s) de risque, veuillez spécifier:
  (Seulement les patients avec des signes cliniques pour une infection, etc…)

Dépistage ciblé à l'admission

2. CPEs

* Pratiquez-vous un <u>dépistage ciblé à l'admission</u> pour les CPEs, en fonction du profil de risque des patients hospitalisés ?

Les écouvillons de dépistage sont ciblés lorsqu'ils sont utilisés sur les patients avec des facteurs de risque uniquement.

- ( ) Oui, dans toute l'institution
- ( ) Non
- ( ) Je ne sais pas
- ( ) Oui, dans une ou plusieurs unité(s):
  (ex: USI, unités de dialyse...)

Pratiques de dépistage des bactéries multirésistantes à l'admission.

Enquête auprès des hôpitaux suisses.

Dépistage ciblé à l'admission

2. CPEs

* Quels sont les facteurs de risque considérés pour le dépistage à l'admission des CPEs ?

- ☐ Je ne sais pas
- ☐ Porteur déjà connu pour une CPE
- ☐ Transfert direct venant d'un hôpital à l'étranger
- ☐ Transfert direct venant d'un hôpital Suisse
- ☐ Transfert direct venant d'un établissement de soin à longue durée ou d'une maison de retraite en Suisse
- ☐ Antécédent d'hospitalisation récente à l'étranger (sans transfert direct)
- ☐ Antécédent de voyage dans un pays endémique pour les BMRs (ex : Inde)
- ☐ Si autre(s) facteur(s) de risque, veuillez spécifier:
  (Seulement les patients avec des signes cliniques pour une infection, etc…)

Pratiques de dépistage des bactéries multirésistantes à l'admission.

Enquête auprès des hôpitaux suisses.

Dépistage ciblé à l'admission

3. MDR-Acinetobacter

* Pratiquez-vous un dépistage ciblé à l'admission pour les MDR-Acinetobacter, en fonction du profil de risque des patients hospitalisés ?

Les écouvillons de dépistage sont ciblés lorsqu'ils sont utilisés sur les patients avec des facteurs de risque uniquement.

- ○ Oui, dans toute l'institution
- ○ Non
- ○ Je ne sais pas
- ○ Oui, dans une ou plusieurs unité(s):
  (ex: USI, unités de dialyse...)

Pratiques de dépistage des bactéries multirésistantes à l'admission.

Enquête auprès des hôpitaux suisses.

Dépistage ciblé à l'admission

3. MDR-Acinetobacter

* Quels sont les facteurs de risque considérés pour le dépistage à l'admission des MDR-Acinetobacter ?

- ☐ Je ne sais pas
- ☐ Porteur déjà connu pour un MDR-Acinetobacter
- ☐ Transfert direct venant d’un hôpital à l’étranger
- ☐ Transfert direct venant d’un hôpital Suisse
- ☐ Transfert direct venant d’un établissement de soin à longue durée ou d’une maison de retraite en Suisse
- ☐ Antécédent d’hospitalisation récente à l’étranger (sans transfert direct)
- ☐ Antécédent de voyage dans un pays endémique pour les BMRs
- ☐ Si autre(s) facteur(s) de risque, veuillez spécifier:
  (Seulement les patients avec des signes cliniques pour une infection, etc…)

Dépistage ciblé à l'admission

4. MDR-Pseudomonas

* Pratiquez-vous un <u>dépistage ciblé à l’admission</u> pour les MDR-Pseudomonas,
en fonction du profil de risque des patients hospitalisés ?

Les écouvillons de dépistage sont ciblés lorsqu’ils sont utilisés sur les patients avec des facteurs de risque uniquement.

- ○ Oui, dans toute l'institution ○ Non ○ Je ne sais pas
- ○ Oui, dans une ou plusieurs unité(s):
  (ex: USI, unités de dialyse...)

Pratiques de dépistage des bactéries multirésistantes à l’admission.

Enquête auprès des hôpitaux suisses.

Dépistage ciblé à l'admission

4. MDR-Pseudomonas

* Quels sont les facteurs de risque considérés pour le dépistage à l'admission des MDR-Pseudomonas ?

- ☐ Je ne sais pas
- ☐ Porteur déjà connu pour un MDR-Pseudomonas
- ☐ Transfert direct venant d'un hôpital à l'étranger
- ☐ Transfert direct venant d'un hôpital Suisse
- ☐ Transfert direct venant d'un établissement de soin à longue durée ou d'une maison de retraite en Suisse
- ☐ Antécédent d'hospitalisation récente à l'étranger (sans transfert direct)
- ☐ Antécédent de voyage dans un pays endémique pour les BMRs
- ☐ Si autre(s) facteur(s) de risque, veuillez spécifier:
  (Seulement les patients avec des signes cliniques pour une infection, etc…)

Dépistage ciblé à l'admission

5. VRE

* Pratiquez-vous un <u>dépistage ciblé à l'admission</u> pour les VRE,
en fonction du profil de risque des patients hospitalisés ?

Les écouvillons de dépistage sont ciblés lorsqu'ils sont utilisés sur les patients avec des facteurs de risque uniquement.

- ○ Oui, dans toute l'institution ○ Non ○ Je ne sais pas
- ○ Oui, dans une ou plusieurs unité(s):
  (ex: USI, unités de dialyse...)

Dépistage ciblé à l'admission

5. VRE

* Quels sont les facteurs de risque considérés pour le dépistage à l'admission des VRE ?

- ☐ Je ne sais pas
- ☐ Porteur déjà connu pour un VRE
- ☐ Transfert direct venant d’un hôpital à l’étranger
- ☐ Transfert direct venant d’un hôpital Suisse
- ☐ Transfert direct venant d’un établissement de soin à longue durée ou d’une maison de retraite en Suisse
- ☐ Antécédent d’hospitalisation récente à l’étranger (sans transfert direct)
- ☐ Antécédent de voyage dans un pays endémique pour les BMRs
- ☐ Si autre(s) facteur(s) de risque, veuillez spécifier:
  (Seulement les patients avec des signes cliniques pour une infection, etc…)

Pratiques de dépistage des bactéries multirésistantes à l’admission.

Enquête auprès des hôpitaux suisses.

Dépistage ciblé à l'admission

6. MRSA

* Pratiquez-vous un dépistage ciblé à l’admission pour les MRSA, en fonction du profil de risque des patients hospitalisés ?

Les écouvillons de dépistage sont ciblés lorsqu’ils sont utilisés sur les patients avec des facteurs de risque uniquement.

- ◯ Oui, dans toute l'institution
- ◯ Non
- ◯ Je ne sais pas
- ◯ Oui, dans une ou plusieurs unité(s): *(ex: USI, unités de dialyse...)*

Pratiques de dépistage des bactéries multirésistantes à l’admission.

Enquête auprès des hôpitaux suisses.

Dépistage ciblé à l'admission

6. MRSA

* Quels sont les facteurs de risque considérés pour le dépistage à l'admission des MRSA ?

- ☐ Je ne sais pas
- ☐ Porteur déjà connu pour un MRSA
- ☐ Transfert direct venant d’un hôpital à l’étranger
- ☐ Transfert direct venant d’un hôpital Suisse
- ☐ Transfert direct venant d’un établissement de soin à longue durée ou d’une maison de retraite en Suisse
- ☐ Antécédent d’hospitalisation récente à l’étranger (sans transfert direct)
- ☐ Antécédent de voyage dans un pays endémique pour les BMRs
- ☐ Si autre(s) facteur(s) de risque, veuillez spécifier: *(Personnel hospitalier au début de leur emploi, seulement les patients avec des signes cliniques pour une infection, etc…)*

Pratiques de dépistage des bactéries multirésistantes à l’admission.

Enquête auprès des hôpitaux suisses.

Dépistage ciblé à l'admission

Questions générales

* Si le transfert direct venant d'un hôpital Suisse est un facteur de risque considéré pour le dépistage ciblé des BMRs à l'admission:

Incluez-vous spécifiquement une région géographique, linguistique ou bien une catégorie d’hôpital en Suisse ?

(Ex : les hôpitaux se trouvant au Tessin, du côté Suisse Romand ou bien les hôpitaux universitaires.)

- ○ Non
- ○ Je ne sais pas
- ○ Oui (veuillez spécifier:)

* Si l'antécédent d’hospitalisation récente à l’étranger (sans transfert direct) est un facteur de risque considéré
pour le dépistage ciblé des BMRs à l'admission:

Quel est le délai considéré entre cette exposition et le dépistage à l’admission du patient après son retour en Suisse?

Pratiques de dépistage des bactéries multirésistantes à l’admission.

Enquête auprès des hôpitaux suisses.

Dépistage universel et/ou ciblé à l'admission

Effectuez-vous des frottis de dépistage pour les BMRs suivantes ?

Les réponses à cette question influenceront les prochaines questions qui vous seront proposées.

	Oui	Non OU BMR non dépistée
E-BLSE	○	○
CPE	○	○
MDR-Acinetobacter et/ou MDR-Pseudomonas	○	○
VRE	○	○
MRSA	○	○

Effectuez-vous également des cultures cliniques pour les BMRs suivantes, en présence de certains symptômes cliniques ?

(Par exemple: expectorations, plaies, urines...)

Les réponses à cette question influenceront les prochaines questions qui vous seront proposées.

	Oui	Non
E-BLSE	○	○
CPE	○	○
MDR-Acinetobacter et/ou MDR-Pseudomonas	○	○
VRE	○	○
MRSA	○	○

Dépistage universel et/ou ciblé à l'admission

* Quel(s) est (sont) le(s) site(s) d'échantillonnage utilisé(s) pour les frottis de dépistage des BMRs suivantes ?

Cocher <u>tous</u> les sites pertinents:

	Narines	Gorge / Pharynx	Région axillaire	Région de l'aine / pli inguinal	Marge anale / Rectum *(Frottis et/ou culture de selles)*	Autre(s) site(s)
E-BLSE	☐	☐	☐	☐	☐	☐
CPE	☐	☐	☐	☐	☐	☐
MDR-Acinetobacter et/ou MDR-Pseudomonas	☐	☐	☐	☐	☐	☐
VRE	☐	☐	☐	☐	☐	☐
MRSA	☐	☐	☐	☐	☐	☐

Autre(s) site(s) et leur BMR correspondante:

* Quelle(s) est (sont) la(les) culture(s) clinique(s) prélevée(s) pour dépister les BMRs à l'admission, en présence de certains symptômes ?

Cocher <u>tous</u> les sites pertinents:

	Plaies (si présentes)	Expectorations et/ou Aspiration trachéale (si présentes)	Urine (en cas de sonde urinaire)	Autre prélèvement
E-BLSE	☐	☐	☐	☐
CPE	☐	☐	☐	☐
MDR-Acinetobacter et/ou MDR-Pseudomonas	☐	☐	☐	☐
VRE	☐	☐	☐	☐
MRSA	☐	☐	☐	☐

Autre(s) prélèvement(s) et leur BMR correspondante:

Dépistage universel et/ou ciblé à l'admission

* Pratiquez-vous un dépistage répété (minimum 2x) de manière routinière pour les patients à plus haut risque de portage de BMR ?
(ex : transfert direct d'un patient resté 10 jours dans une USI en Grèce, Italie, Maroc…)

○ Oui ○ Non ○ Je ne sais pas

Pratiques de dépistage des bactéries multirésistantes à l'admission.

Enquête auprès des hôpitaux suisses.

Mesures de contact préemptives

* Implémentez-vous les mesures de contact de manière préemptive pour les patients à haut risque, transférés de l'étranger, en attendant les résultats de laboratoire suite au dépistage à l'admission ?
Si absence d'USI dans votre établissement, cochez "non-applicable"

	Pas de mesure spécifique	Précautions de contact *(chambre commune)*	Précautions de contact *(chambre individuelle obligatoire)*	Non-applicable
USI	○	○	○	○
Autre(s) service(s)	○	○	○	○

Si d'autres services sont concernés, veuillez les nommer:

[ ]

Pratiques de dépistage des bactéries multirésistantes à l'admission.

Enquête auprès des hôpitaux suisses.

Implémentation et adhérence au dépistage de routine

* Avez-vous des guidelines locales ou recommandations écrites disponibles pour votre personnel hospitalier, détaillant la procédure du dépistage à l'admission des BMRs ?

○ Oui ○ Non ○ Je ne sais pas

Implémentation et adhérence au dépistage de routine

* Faites-vous face à un ou plusieurs problèmes pour implémenter le dépistage des BMRs à l'admission?

Si oui, indiquer ce(s) problème(s) et obstacle(s).

(Cocher <u>tous</u> les problèmes pertinents)

- ☐ Non-adhérence du personnel hospitalier
- ☐ Le personnel n'a pas assez de temps pour pratiquer le dépistage à l'admission
- ☐ Difficulté pour identifier les patients à risque
- ☐ Problème de remboursement
- ☐ Manque de soutien informatique
- ☐ Pas de laboratoire de microbiologie disponible sur le site
- ☐ Problème éthique
- ☐ Très faible prévalence des BMRs dans notre établissement
- ☐ Un résultat positif ne changera pas notre prise en charge
- ☐ Ces problèmes n'ont jamais été considérés jusqu'à maintenant
- ☐ Autre (préciser)

Avez-vous d'autres suggestions pour homogénéiser les pratiques de dépistage pour les BMRs à l'admission dans les hôpitaux en Suisse ? (optionnel)

Autre commentaire ou suggestion: (optionnel)

Nous vous sommes reconnaissants de votre participation.

Si vous avez des questions ou besoin d'une assistance pour remplir ce questionnaire, prière de nous contacter:

**Personne de contact: Romain Martischang**
**Téléphone: 022 372 98 97**<br>**Lundi - Vendredi**<br>**08:30 – 17:30**
**Adresse mail: romain.martischang@hcuge.ch**

Veuillez agréer, Madame, Monsieur, l'expression de nos sentiments les plus respectueux,

Division Maladies transmissibles
Le responsable de la division
Dr. med. Daniel Koch

Swissnoso
Le Président
Professeur Andreas F. Widmer

HUG
Le responsable de l'enquête
Professeur Stephan Harbarth
