# Supplementary material for: Nation-wide survey of screening practices to detect carriers of multi-drug resistant organisms upon admission to Swiss healthcare institutions
Source: Antimicrob Resist Infect Control. 2019 Feb 13;8:37. doi: 10.1186/s13756-019-0479-5 (PMC6375162; doi:10.1186/s13756-019-0479-5)
Supplement: Supplementary file 2 — Online survey German. (PDF 348 kb) [file 13756_2019_479_MOESM2_ESM.pdf]

Aktuelle Praxis beim Eintritts-Screening für multiresistente Mikroorganismen in Schweizer Spitälern

Schweizerische Eidgenossenschaft
Confédération suisse
Confederazione Svizzera
Confederaziun svizra

Département fédéral de l'intérieur DFI
Office fédéral de la santé publique OFSP

Anleitung:

- Dieser Fragebogen sollte von einer Person ausgefüllt werden, die mit der Infektionsprävention in Ihrer Einrichtung vertraut ist.

- Die Umfrage sollte nicht mehr als 15 Minuten Ihrer Arbeitszeit in Anspruch nehmen und ist zur Erarbeitung von Screening-Leitlinien für multiresistente Bakterien (MRB) in der Schweiz äusserst wichtig.

Bitte beantworten Sie alle Fragen.
Ihre Antworten werden absolut vertraulich behandelt.
Falls Sie Fragen haben oder Hilfe beim Ausfüllen des Fragebogens benötigen, können Sie folgende Nummer anrufen:
Kontaktperson: Romain Martischang
Telefon: 022 372 98 97<br>Montag bis Freitag<br>08:30 – 17:30
E-mail: romain.martischang@hcuge.ch
Ihre Teilnahme an dieser Befragung ist für den Erfolg des Projekts entscheidend!
VIELEN DANK FÜR IHRE MITHILFE

Aktuelle Praxis beim Eintritts-Screening für

multiresistente Mikroorganismen in Schweizer Spitälern

Zusatzinformationen:

Praktische Anweisungen:

Um in diesem Fragebogen fortzufahren, klicken Sie auf "Weiter", um zurück zu gehen, klicken Sie auf "Zurück". Bitte benutzen Sie nicht die Pfeiltasten in Ihrem Browser.

Wenn möglich, füllen Sie diesen Fragebogen in einem Anlauf aus. Ihre Antworten werden jedoch jedes Mal gespeichert, sobald eine neue Seite angezeigt wird. Sie haben die Möglichkeit, diesen Fragebogen zu schließen, um ihn später wieder aufzunehmen, allerdings unter folgenden Bedingungen:

1) Verwenden Sie den gleichen Computer und den gleichen Browser, (Google Chrome, Internet Explorer ...), um diesen Fragebogen wieder aufzunehmen.
2) Löschen Sie nicht Ihre Browsereinträge.
(Den Cache mit den registrierten Cookies Ihres Browsers.)

Abkürzungen von MultiResistenten Bakterien (MRB):

- ESBL-PE: *Extended-Spectrum Beta-Lactamasen (ESBL) produzierende Enterobakterien, insbesondere ESBL-produzierende Klebsiella und/oder ESBL-produzierende E.coli*
- CPE: *Carbapenemase-produzierende Enterobakterien*
- MDR-Acinetobacter oder MDR-Pseudomonas: *Acinetobacter baumannii bzw. Pseudomonas aeruginosa, die gegen mindestens ein Antibiotikum in drei oder mehr Klassen antimikrobieller Substanzen resistent sind.*
- MRSA: *Methicillin-resistenter Staphylococcus aureus*
- VRE: *Vancomycin-resistente Enterokokken.*

Aktuelle Praxis beim Eintritts-Screening für

multiresistente Mikroorganismen in Schweizer Spitälern

Epidemiologische Daten

* Personenbezogene Daten:

Name, Vorname:

E-Mail-Adresse :

* Ihre Funktion:

* Berufsbezogene Daten:

Spitalname:

Stadt:

Kanton:

* Spitalkategorie:

- ○ öffentlich
- ○ privat

* Art der stationären Behandlungen in Ihrer Einrichtung:

(mehrere Antworten möglich)

- ☐ Akutpflege
- ☐ Langzeitpflege und Rehabilitation
- ☐ Pädiatrie
- ☐ Psychiatrie
- ☐ Andere:

* Wie viele Akutbetten hat Ihre Gesundheitseinrichtung ?

- ○ < 200
- ○ 200 - 500
- ○ > 500
- ○ Weiss nicht

* Intensivstation vorhanden:

(Die Intermediate Care zählt nicht als Intensivstation)

- ○ Ja
- ○ Nein

* Wird in Ihrer Einrichtung ein Eintrittsscreening durchgeführt für mindestens eine Kategorie von MRB ?

(Entweder generell oder nur auf bestimmte Risikopatienten abzielend)

◯ Ja ◯ Nein

Aktuelle Praxis beim Eintritts-Screening für multiresistente Mikroorganismen in Schweizer Spitälern

Generelles Eintrittsscreening

* Gibt es in Ihrem Spital eine Abteilung, in der Sie ein Generelles Eintrittsscreening bei allen eintretenden Patienten routinemässig durchführen, unabhängig von einem bestimmten Risikoprofil?

	Ja	Nein	Weiss nicht
Nur Intensivstation	◯	◯	◯
Andere Abteilungen *(Chirurgie, Geriatrie...)*	◯	◯	◯

Benennen Sie diese anderen Abteilungen:

[ ]

Aktuelle Praxis beim Eintritts-Screening für multiresistente Mikroorganismen in Schweizer Spitälern

Generelles Eintrittsscreening

* Welche Arten von MRB werden in den betroffenen Abteilungen durch generelles Eintrittsscreening aller Patienten gesucht?

	ESBL-PE	CPE	MDR-Acinetobacter	MDR-Pseudomonas	VRE	MRSA
Nur Intensivstation	☐	☐	☐	☐	☐	☐
Andere Abteilungen *(Chirurgie, Geriatrie...)*	☐	☐	☐	☐	☐	☐

Aktuelle Praxis beim Eintritts-Screening für multiresistente Mikroorganismen in Schweizer Spitälern

Gezieltes Eintrittsscreening

* Führen Sie gezieltes Eintrittsscreening für MRB bei Hochrisikopatienten durch, die in Ihr Spital eintreten?

Screening-Abstriche gelten dann als gezielt, wenn sie bei Patienten mit spezifischen Risikofaktoren durchgeführt werden.

○ Ja ○ Nein ○ Weiss nicht

Aktuelle Praxis beim Eintritts-Screening für multiresistente Mikroorganismen in Schweizer Spitälern

Gezieltes Eintrittsscreening

1. ESBL-PE

* Führen Sie gezieltes Eintrittsscreening für ESBL-PE bei Hochrisikopatienten durch, die in Ihr Spital eintreten?

Screening-Abstriche gelten dann als gezielt, wenn sie bei Patienten mit spezifischen Risikofaktoren durchgeführt werden.

○ Ja, in der ganzen Einrichtung ○ Nein ○ Weiss nicht

○ Ja, in ausgewählten Abteilungen:
(z.B. Intensivstation, Dialyse...)

Aktuelle Praxis beim Eintritts-Screening für multiresistente Mikroorganismen in Schweizer Spitälern

Gezieltes Eintrittsscreening

2. ESBL-PE

* Welche Risikofaktoren werden berücksichtigt, um gezielte ESBL-PE Abstrichuntersuchungen zu veranlassen?

- [ ] Weiss nicht
- [ ] Bekannter früherer Träger eines ESBL-PE
- [ ] Direktüberweisung aus einem Spital im Ausland
- [ ] Direktüberweisung aus einem Spital in der Schweiz
- [ ] Direktüberweisung aus einem Alters- oder Pflegeheim in der Schweiz
- [ ] Kürzliche Hospitalisation im Ausland (ohne Direktüberweisung)
- [ ] Kürzliche Reise in Land mit hyperendemischen MRB
- [ ] Falls andere Risikofaktoren berücksichtigt werden, bitte hier nennen: *(Zum Beispiel nur Patienten mit Anzeichen einer klinischen Infektion usw.)*

Aktuelle Praxis beim Eintritts-Screening für

multiresistente Mikroorganismen in Schweizer Spitälern

Gezieltes Eintrittsscreening

2. CPE

* Führen Sie gezieltes Eintrittsscreening für CPE bei Hochrisikopatienten durch, die in Ihr Spital eintreten?

Screening-Abstriche gelten dann als gezielt, wenn sie bei Patienten mit spezifischen Risikofaktoren durchgeführt werden.

○ Ja, in der ganzen Einrichtung ○ Nein ○ Weiss nicht

○ Ja, in ausgewählten Abteilungen: *(z.B. Intensivstation, Dialyse...)*

Aktuelle Praxis beim Eintritts-Screening für

multiresistente Mikroorganismen in Schweizer Spitälern

Gezieltes Eintrittsscreening

2. CPE

* Welche Risikofaktoren werden berücksichtigt, um gezielte CPE Abstrichuntersuchungen zu veranlassen?

- [ ] Weiss nicht
- [ ] Bekannter früherer Träger eines CPE
- [ ] Direktüberweisung aus einem Spital im Ausland
- [ ] Direktüberweisung aus einem Spital in der Schweiz
- [ ] Direktüberweisung aus einem Alters- oder Pflegeheim in der Schweiz
- [ ] Kürzliche Hospitalisation im Ausland (ohne Direktüberweisung)
- [ ] Kürzliche Reise in Land mit hyperendemischen MRB (z.B. Indien)
- [ ] Falls andere Risikofaktoren berücksichtigt werden, bitte hier nennen: *(Zum Beispiel nur Patienten mit Anzeichen einer klinischen Infektion usw.)*

Gezieltes Eintrittsscreening

3. MDR-Acinetobacter

* Führen Sie gezieltes Eintrittsscreening für MDR-Acinetobacter bei Hochrisikopatienten durch, die in Ihr Spital eintreten?

Screening-Abstriche gelten dann als gezielt, wenn sie bei Patienten mit spezifischen Risikofaktoren durchgeführt werden.

- ○ Ja, in der ganzen Einrichtung ○ Nein ○ Weiss nicht
- ○ Ja, in ausgewählten Abteilungen: *(z.B. Intensivstation, Dialyse...)*

Gezieltes Eintrittsscreening

3. MDR-Acinetobacter

* Welche Risikofaktoren werden berücksichtigt, um gezielte MDR-Acinetobacter Abstrichuntersuchungen zu veranlassen?

- [ ] Weiss nicht
- [ ] Bekannter früherer Träger eines MDR-Acinetobacter
- [ ] Direktüberweisung aus einem Spital im Ausland
- [ ] Direktüberweisung aus einem Spital in der Schweiz
- [ ] Direktüberweisung aus einem Alters- oder Pflegeheim in der Schweiz
- [ ] Kürzliche Hospitalisation im Ausland (ohne Direktüberweisung)
- [ ] Kürzliche Reise in Land mit hyperendemischen MRB
- [ ] Falls andere Risikofaktoren berücksichtigt werden, bitte hier nennen: *(Zum Beispiel nur Patienten mit Anzeichen einer klinischen Infektion usw.)*

Aktuelle Praxis beim Eintritts-Screening für

multiresistente Mikroorganismen in Schweizer Spitälern

Gezieltes Eintrittsscreening

4. MDR-Pseudomonas

* Führen Sie gezieltes Eintrittsscreening für MDR-Pseudomonas bei Hochrisikopatienten durch, die in Ihr Spital eintreten?

Screening-Abstriche gelten dann als gezielt, wenn sie bei Patienten mit spezifischen Risikofaktoren durchgeführt werden.

- ○ Ja, in der ganzen Einrichtung
- ○ Nein
- ○ Weiss nicht
- ○ Ja, in ausgewählten Abteilungen: *(z.B. Intensivstation, Dialyse...)*

Gezieltes Eintrittsscreening

4. MDR-Pseudomonas

* Welche Risikofaktoren werden berücksichtigt, um gezielte MDR-Pseudomonas Abstrichuntersuchungen zu veranlassen?

- ☐ Weiss nicht
- ☐ Bekannter früherer Träger eines MDR-Pseudomonas
- ☐ Direktüberweisung aus einem Spital im Ausland
- ☐ Direktüberweisung aus einem Spital in der Schweiz
- ☐ Direktüberweisung aus einem Alters- oder Pflegeheim in der Schweiz
- ☐ Kürzliche Hospitalisation im Ausland (ohne Direktüberweisung)
- ☐ Kürzliche Reise in Land mit hyperendemischen MRB
- ☐ Falls andere Risikofaktoren berücksichtigt werden, bitte hier nennen: *(Zum Beispiel nur Patienten mit Anzeichen einer klinischen Infektion usw.)*

Aktuelle Praxis beim Eintritts-Screening für

multiresistente Mikroorganismen in Schweizer Spitälern

Gezieltes Eintrittsscreening

5. VRE

* Führen Sie gezieltes Eintrittsscreening für VRE bei Hochrisikopatienten durch, die in Ihr Spital eintreten?

Screening-Abstriche gelten dann als gezielt, wenn sie bei Patienten mit spezifischen Risikofaktoren durchgeführt werden.

◯ Ja, in der ganzen Einrichtung ◯ Nein ◯ Weiss nicht

◯ Ja, in ausgewählten Abteilungen:
(z.B. Intensivstation, Dialyse...)

Aktuelle Praxis beim Eintritts-Screening für

multiresistente Mikroorganismen in Schweizer Spitälern

Gezieltes Eintrittsscreening

5. VRE

* Welche Risikofaktoren werden berücksichtigt, um gezielte VRE Abstrichuntersuchungen zu veranlassen?

☐ Weiss nicht

☐ Bekannter früherer Träger eines VRE

☐ Direktüberweisung aus einem Spital im Ausland

☐ Direktüberweisung aus einem Spital in der Schweiz

☐ Direktüberweisung aus einem Alters- oder Pflegeheim in der Schweiz

☐ Kürzliche Hospitalisation im Ausland
(ohne Direktüberweisung)

☐ Kürzliche Reise in Land mit hyperendemischen MRB

☐ Falls andere Risikofaktoren berücksichtigt werden, bitte hier nennen:
(Zum Beispiel nur Patienten mit Anzeichen einer klinischen Infektion usw.)

Aktuelle Praxis beim Eintritts-Screening für

multiresistente Mikroorganismen in Schweizer Spitälern

Gezieltes Eintrittsscreening

6. MRSA

* Führen Sie gezieltes Eintrittsscreening für MRSA bei Hochrisikopatienten durch, die in Ihr Spital eintreten?

Screening-Abstriche gelten dann als gezielt, wenn sie bei Patienten mit spezifischen Risikofaktoren durchgeführt werden.

○ Ja, in der ganzen Einrichtung ○ Nein ○ Weiss nicht

○ Ja, in ausgewählten Abteilungen:
(z.B. Intensivstation, Dialyse...)

Aktuelle Praxis beim Eintritts-Screening für

multiresistente Mikroorganismen in Schweizer Spitälern

Gezieltes Eintrittsscreening

6. MRSA

* Welche Risikofaktoren werden berücksichtigt, um gezielte MRSA Abstrichuntersuchungen zu veranlassen?

- [ ] Weiss nicht
- [ ] Bekannter früherer Träger eines MRSA
- [ ] Direktüberweisung aus einem Spital im Ausland
- [ ] Direktüberweisung aus einem Spital in der Schweiz
- [ ] Direktüberweisung aus einem Alters- oder Pflegeheim in der Schweiz
- [ ] Kürzliche Hospitalisation im Ausland (ohne Direktüberweisung)
- [ ] Kürzliche Reise in Land mit hyperendemischen MRB
- [ ] Falls andere Risikofaktoren berücksichtigt werden, bitte hier nennen: *(Zum Beispiel Gesundheitspersonal bei Einstellung, nur Patienten mit Anzeichen einer klinischen Infektion usw.)*

Aktuelle Praxis beim Eintritts-Screening für multiresistente Mikroorganismen in Schweizer Spitälern

Gezieltes Eintrittsscreening

Allgemeine Fragen:

* Falls die Direktüberweisung von einem Spital in der Schweiz ein Risikofaktor für das gezielte Eintritts-Screening in Ihrer Einrichtung darstellt:

Unterscheiden Sie dabei innerhalb der Schweiz nach bestimmten geografischen Regionen oder Sprachregionen oder einer Spitalart?

(z.B.: Tessiner oder Westschweizer Spital; Universitätsspital…)

- ◯ Nein ◯ Weiss nicht
- ◯ Ja (bitte genauer angeben)

* Nur falls Sie die betreffende Frage (kürzlicher Spitalaufenthalt im Ausland des Patienten, ohne Direktttransfer) für mindestens eine MRB mit Ja beantwortet haben:

In welchem Zeitraum nach der Rückkehr in die Schweiz wird ein Eintritts-Screening durchgeführt?

Aktuelle Praxis beim Eintritts-Screening für multiresistente Mikroorganismen in Schweizer Spitälern

Generelles und/oder gezieltes Eintrittsscreening

Veranlassen Sie Abstrichuntersuchungen für die folgenden MRB ?

Die Antworten auf diese Frage beeinflussen die nächste Fragenauswahl.

	Ja	Nein oder MRB wird nicht gesucht
ESBL-PE	○	○
CPE	○	○
MDR-Acinetobacter und/oder MDR-Pseudomonas	○	○
VRE	○	○
MRSA	○	○

Bei Vorliegen entsprechender klinischer Symptome, veranlassen Sie zusätzliche bakteriologische Untersuchungen (z.B. Urin, Sputum, Wunden) ?

Die Antworten auf diese Frage beeinflussen die nächste Fragenauswahl.

	Ja	Nein
ESBL-PE	○	○
CPE	○	○
MDR-Acinetobacter und/oder MDR-Pseudomonas	○	○
VRE	○	○
MRSA	○	○

Generelles und/oder gezieltes Eintrittsscreening

* An welchen Körperstellen führen Sie bei Eintritt-Screenings die Abstrichuntersuchungen für spezifische MRB durch?

Bitte alle zutreffenden Stellen ankreuzen:

	Nase	Rachen	Axilla	Leiste/Inguinal	Rektal *(Abstrich oder Stuhlkultur)*	Andere Körperstelle
ESBL-PE	☐	☐	☐	☐	☐	☐
CPE	☐	☐	☐	☐	☐	☐
MDR-Acinetobacter und/oder MDR-Pseudomonas	☐	☐	☐	☐	☐	☐
VRE	☐	☐	☐	☐	☐	☐
MRSA	☐	☐	☐	☐	☐	☐

Andere Körperstelle:

* Bei Vorliegen klinischer Symptome, welche zusätzlichen bakteriologischen Kulturen veranlassen Sie durchzuführen?

Bitte alle zutreffenden Stellen ankreuzen:

	Wunde (falls vorhanden)	Sputum oder Trachealsekret (falls vorhanden)	Urin (bei Harnwegs-katheter)	Andere Körperstelle
ESBL-PE	☐	☐	☐	☐
CPE	☐	☐	☐	☐
MDR-Acinetobacter und/oder MDR-Pseudomonas	☐	☐	☐	☐
VRE	☐	☐	☐	☐
MRSA	☐	☐	☐	☐

Andere Körperstelle:

[ ]

Aktuelle Praxis beim Eintritts-Screening für multiresistente Mikroorganismen in Schweizer Spitälern

Generelles und/oder gezieltes Eintrittsscreening

* Führen Sie routinemässig ein wiederholtes Screening (mind. 2x) für Patienten mit dem höchsten Risiko einer MRB-Trägerschaft durch?

(z.B. Direktüberweisung von Patienten, die 10 Tage auf einer Intensivstation in Griechenland, Italien oder Marokko waren)

○ Ja ○ Nein ○ Weiss nicht

Aktuelle Praxis beim Eintritts-Screening für multiresistente Mikroorganismen in Schweizer Spitälern

Präemptive Kontaktisolierung

* Setzen Sie präemptive Kontaktisolations-Massnahmen für Hochrisikopatienten ein, die aus dem Ausland überwiesen werden, bis die Laborergebnisse des Eintritt-Screenings vorliegen?

Wenn es keine Intensivstation in Ihrer Einrichtung gibt, wählen Sie "Nicht zutreffend".

	Keine besonderen Massnahmen	Kontaktisolation *(Mehrbettzimmer möglich)*	Kontaktisolation *(Einzelzimmer zwingend)*	Nicht zutreffend
Intensivstation	◯	◯	◯	◯
Andere Abteilungen	◯	◯	◯	◯

Benennen Sie diese anderen Abteilungen:

Aktuelle Praxis beim Eintritts-Screening für

multiresistente Mikroorganismen in Schweizer Spitälern

Routine-Screening: Durchführung und Adhärenz

* Haben Sie lokale Richtlinien oder Empfehlungen für Ihr Spitalpersonal, in denen die MRB-Screening-Kriterien beim Eintritt festgehalten sind ?

◯ Ja ◯ Nein ◯ Weiss nicht

Aktuelle Praxis beim Eintritts-Screening für

multiresistente Mikroorganismen in Schweizer Spitälern

Routine-Screening: Durchführung und Adhärenz

* Gibt es manchmal Probleme mit dem MRB-Eintritts-Screening?

Bitte geben Sie den Grund/die Gründe dafür an.

(bitte alle zutreffenden Antworten ankreuzen)

- ☐ Gesundheitspersonal hält sich nicht daran
- ☐ Personal hat nicht genügend Zeit, das Eintritts-Screening durchzuführen
- ☐ Schwierigkeit, Patienten mit Risikofaktoren zu identifizieren
- ☐ Probleme mit der Vergütung
- ☐ Mangelnde Unterstützung der Informatiker
- ☐ Kein Mikrobiologie-Labor vor Ort
- ☐ Ethische Fragen
- ☐ Sehr niedrige MRB-Prävalenz in unserer Einrichtung
- ☐ Positives Ergebnis hätte keinen Einfluss auf das Management
- ☐ Bisher nie erwogen
- ☐ Anderes, Bitte angeben:

Haben Sie Vorschläge, wie die MRB-Screening-Praxis in der Schweiz vereinheitlicht werden könnte? (optional)

Andere Kommentare und Vorschläge (optional)

Aktuelle Praxis beim Eintritts-Screening für multiresistente Mikroorganismen in Schweizer Spitälern

Wir danken Ihnen für Ihre Teilnahme.

Für Fragen wenden Sie sich bitte an:

**Kontaktperson: Romain Martischang**
**Telefon: 022 372 98 97**<br>**Montag bis Freitag**<br>**08:30 – 17:30**
**E-Mail: romain.martischang@hcuge.ch**

Freundliche Grüsse

Abteilung Übertragbare Krankheiten
Abteilungsleiter
Dr. med. Daniel Koch

Swissnoso
Präsident
Prof. Andreas F. Widmer

HUG
Leiter der Umfrage
Prof. Stephan Harbarth
